# A survey of knowledge-to-action pathways of aging policies and programs in the Arab region: the role of institutional arrangements

**DOI:** 10.1186/s13012-015-0360-8

**Published:** 2015-12-11

**Authors:** Anthony Rizk, Nabil M. Kronfol, Suzanne Moffatt, Shahaduz Zaman, Souha Fares, Abla Mehio Sibai

**Affiliations:** Department of Epidemiology and Population Health, Faculty of Health Sciences, American University of Beirut, Beirut, Lebanon; Lebanese Health Care Management Association, Beirut, Lebanon; Center for Studies on Aging (CSA), Beirut, Lebanon; Institute of Health & Society, Newcastle University, Newcastle, UK; Rafic Hariri School of Nursing, American University of Beirut, Beirut, Lebanon

**Keywords:** Knowledge translation, Institutional arrangements, Aging, Arab region

## Abstract

**Background:**

While population aging challenges Arab governments to ensure well-being in old age, knowledge translation is gaining support worldwide in evidence-based policymaking and service provision. This study examines the status of existing knowledge translation efforts of aging-related research in Arab countries and evaluates the additional role that institutional arrangements (such as ministry departments, national committees, etc.) play in the relationship between knowledge creation and social and health policies and programs.

**Methods:**

Data were triangulated from two regional surveys and a supplementary desk review of academic, civil society, ministry, and UN documents. Using a set of indicators, standardized summative indices (out of 100) were generated for five constructs, namely knowledge creation, institutional arrangements, knowledge translation, and health and social policies and programs. Correlations were assessed using Spearman’s rank correlation (*r*_s_), and bootstrap multiple linear regression models were used.

**Results:**

Arab countries scored highest on the institutional arrangements index (median = 69.5), followed by the knowledge creation index (median = 45.9), and lowest on the knowledge translation index (median = 30.2). Both institutional arrangements and knowledge creation significantly correlated with social and health policies and programs. However, when adjusted for knowledge translation, only institutional arrangements retained a significant association with both outcomes (*r*_s_ = 0.63, *p* value =0.009 and *r*_s_ = 0.69, *p* value =0.01, respectively). Adjusting for institutional arrangements and knowledge creation, the association of knowledge translation with social and health policies and programs was attenuated and non-significant (*r*_s_ = 0.08, *p* value =0.671 and *r*_s_ = 0.12, *p* value =0.634, respectively).

**Conclusions:**

There are two key messages from this study. Firstly, institutional arrangements play a central role in aging social and health policy and program development in the Arab region. Secondly, knowledge translation paradigms in Arab countries may be deficient in factors pertinent for promoting evidence-based decision-making and policy-relevant research. These findings call for the need of strengthening institutional arrangements on aging and for promoting knowledge production that meets policy-relevant priorities.

**Electronic supplementary material:**

The online version of this article (doi:10.1186/s13012-015-0360-8) contains supplementary material, which is available to authorized users.

## Background

Population aging has been acknowledged as a priority issue since the first World Assembly on Aging convened by the United Nations in 1982. Older people are increasing both in number and proportion worldwide, but at a higher pace in developing regions, including the Arab countries. Demographic changes in the region are the result of declining fertility from an average of six children per woman in 1980 to approximately three in 2010, falling mortality by half during this period, and substantial gains in life expectancy at birth for both males and females [1]. While the current proportion of persons older than 65 does not exceed 4 % [2]—in contrast to 11 % in the European Union [3]—projections herald rapid population aging over the next few decades with a threefold increase in the percentage of older people to 12 % in 2050, and exceeding 15 % in 10 out of the 22 Arab countries [1, 4].

The above demographic transitions challenge Arab countries to invest in economic and social development targeted at ensuring well-being in old age. However, Arab countries are heterogeneous in their natural resources, economic systems, political regimes, and national priorities which impact responses to population aging [5]. This situation is exemplified in the existing disparities in retirement benefits across Arab countries, where benefits range from generous pension schemes in high-income and some middle-income countries (e.g., Kuwait and Tunisia) to low and basic benefits in most others [6]. Such economic disparities, among other sociopolitical inequities, have contributed towards and been sharpened by the political transformations that have spread across a number of Arab countries since 2010 (in Tunisia, Egypt, Libya, Syria, and Yemen) as well as by the ongoing conflicts in Iraq, Palestine, Somalia, and the Sudan [7]. Consequently, it is imperative that local research aligns with national sociopolitical priorities and policies and service delivery be based on rigorous context-specific research evidence.

Meanwhile, knowledge translation is gaining support worldwide in evidence-based policymaking and service provision and has been widely endorsed by the World Health Assembly and the World Health Organization [8–10]. The “knowledge-to-action gap” is defined by Graham and colleagues as a deficiency in translating research knowledge to practice (the “Know-Do Gap”), resulting from a lack of linkages between researchers and policymakers [11]. While sociological investigations of this deficiency can be traced back to the 1970s, knowledge translation has been widely investigated since the turn of the century as scholarship that addresses the “ongoing and iterative process and strategy that requires the active and conscious participation of both researchers and research users” in order to accelerate “the natural transformation of knowledge into use” [12]. This acceleration has become increasingly pertinent in modern times due to large investments in creating knowledge that is often underused and not adequately translated into improved policies and programs [13]. Kitson and Bisby trace knowledge translation to an emerging paradigm shift in the way the public becomes active in the knowledge production process [14]. As such, this necessitates research production that meets the needs of society at large and veers away from the traditional “curiosity-driven” route. In order to ensure a systematic flow of generated knowledge into policy and practice, the two-way linkages between stakeholders require new discursive and non-hierarchal structures of decision-making and priority-setting that are inclusive to policymakers, researchers, and civil society groups [14, 15].

Successful knowledge translation efforts worldwide include the Canadian Health Services Research Foundation and the Canadian Institute of Health Research which focus on strengthening evidence-based policies and programs of the national health-care system [12]. In the Arab region, budding knowledge translation efforts are still nascent and include the Knowledge to Policy (K2P) Center [16] at the American University of Beirut and MedCHAMPS, an international network that has conducted policy-directed research on cardiovascular disease and diabetes in the Middle East and North Africa [17]. However, such efforts remain limited in the region due to constraints in human resources, institutional infrastructure and financing, and in many countries, underdeveloped health research capacity [18].

Studies on knowledge translation in the field of aging are scarce worldwide [19, 20], and literature has mostly been non-specific, focusing on issues of structures, processes, and communications for bridging the gap between researchers and policymakers [20–24]. In the Arab region, a number of studies have investigated knowledge translation structures and the knowledge-to-action gap in health systems [25–28]. Findings emphasize the complexity of policymaking and identify several barriers to the use of evidence including scarce financial resources, lack of policy-relevant research as well as corruption, and weak governmental agencies and institutional arrangements [27]. Using data from a regional mapping survey conducted in 16 Arab countries, this study aims to examine the status of existing knowledge translation efforts in the field of aging and evaluates how institutional arrangements and knowledge creation interrelate in the process of knowledge translation to inform evidence-based aging-related social and health policies and programs in the Arab region.

## Methods

### Field work and data collection

Data for this study were drawn and triangulated from two surveys and a supplementary desk review. The first survey, the Madrid International Plan of Action on Aging (MIPAA) Regional Mapping Survey, was conducted by the Center for Studies on Aging (CSA, Lebanon) in partnership with the United Nations Population Fund (UNFPA)-Lebanon and the Economic and Social Commission of Western Asia (ESCWA) in 2012 and the second survey, the “International Conference on Population and Development (ICPD) Beyond 2014 Global Survey,” was conducted by ESCWA in 2012. Both surveys were conducted in preparation for the Regional Population and Development Conference for the Arab States in Egypt in June 2013. The supplementary desk review included academic, civil society, ministry, and UN documents and had a dual purpose: to supplement missing information and to crosscheck data provided in the surveys, as far as information is publicly available. The MIPAA questionnaire [29] benefited from two resources: firstly, the questionnaire developed by HelpAge International (HAI) for the analysis of the global situation 8 years after MIPAA [30] and secondly, the knowledge translation framework on aging and health developed by the WHO [31]. The mapping tool was tailored to the Arab region and was reviewed by an expert group committee consisting of 16 specialists from Lebanon, Jordan, Tunisia, and Egypt as well as senior advisors from UNFPA and ESCWA. The final version of the questionnaire included 96 questions that comprehensively map six areas of the aging agenda: institutional arrangements, research and data, policies and plans of action, older people and development, health and well-being, and enabling and supportive environments [29]. The mapping tool was delivered to country investigators in Arab countries in both English and Arabic. Country investigators were selected by UNFPA country representatives based on their expertise and access to required information and were tasked with conducting interviews with representatives of governmental and non-governmental agencies and retrieving relevant documents and reports in order to complete the mapping survey. Rigorous follow-up was conducted by the research coordinators, and questionnaires were returned electronically.

A total of 22 countries were targeted, and 16 responses were received from country investigators in Algeria, Bahrain, Djibouti, Egypt, Iraq, Jordan, Lebanon, Libya, Morocco, Oman, Palestine, Qatar, Somalia (limited to the province of Somaliland), Sudan, Syria, and Yemen with no response from Comoros, Kuwait, Mauritania, Saudi Arabia, Tunisia, and the United Arab Emirates. The completion rate of the country mapping was high (>75 % of questionnaire items) in Algeria, Bahrain, Djibouti, Iraq, Jordan, Lebanon, Libya, Morocco, Palestine, Qatar, Sudan, and Yemen; average (50–70 %) in Egypt, Oman, and Syria; and low (<40 %) in Somalia. The data collection spanned a 6-month period from July to December 2012. The results of the MIPAA survey were complemented with data from the ICPD survey and an extensive desk review of scientific and gray literature. The ICPD survey included 11 questions focusing on institutional arrangements, policies and programs, civil society organizations, and private sector partners in 18 Arab countries (surveys not received from Djibouti, Libya, Saudi Arabia, and the United Arab Emirates).

### Framework

**Fig. 1:**
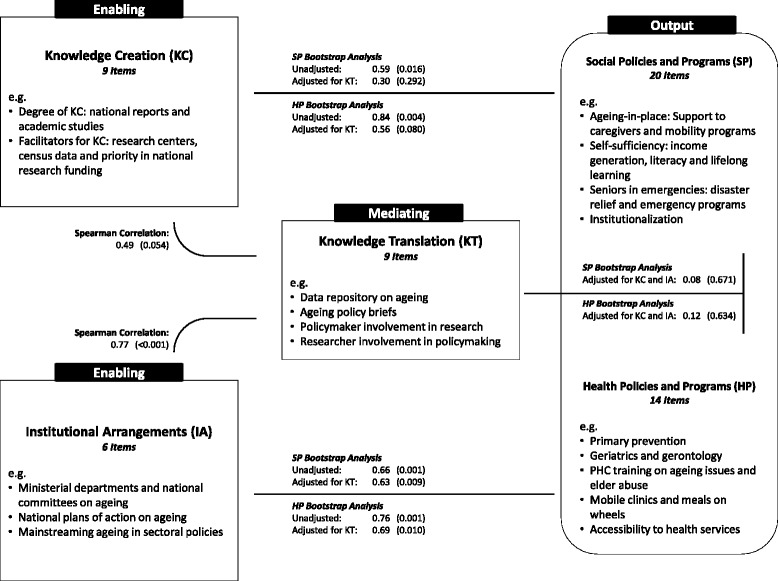
Conceptual framework

The framework utilizes five distinct constructs as can be seen in Fig. 1. The knowledge translation construct (KT) was adapted from the knowledge translation framework on aging and health recently developed by the WHO [31]. The remaining four constructs, namely knowledge creation (KC), institutional arrangements (IA), and social and health policies and programs (SP and HP, respectively) were adapted from the MIPAA [32]. Through using these constructs, this study builds on the “knowledge-to-action” framework developed by Graham et al. [11] where “knowledge” is represented by the KC which includes both the presence of and drivers for empirical research and “action” is represented by the SP and HP as activities where knowledge is applied [11, 31]. This study introduces the IA into the knowledge-to-action framework, where IA refers to the presence and effectiveness of formal decision-making bodies such as ministry departments and national committees on aging. These five distinct constructs were operationalized into indices using several indicators of the yes/no, five-point Likert scale, and open-ended type of questions, listed in the Additional file 1. While the relationships between the constructs are envisioned as non-linear and multidirectional rather than causal, we examine in this study the role of IA and KC as enabling factors that influence SP and HP ; while KT is treated  as a mediating variable in the relationship between the enabling factors (IA and KC) and the outcomes (SP and HP).

Policies and programs, the outcome variables, were assessed in both social and health domains through 20 and 14 indicators, respectively. The SP index combined a wide range of indicators spanning three domains: aging-in-place, self-sufficiency of older persons, and institutionalization. The HP index comprised of ten indicators on the presence of geriatric and palliative care services, elderly homes, and mobile clinics, among others. The KC index was evaluated through nine indicators that assessed knowledge production and its facilitating factors. The IA index was evaluated through six items on departments, national commissions, and plans of action on aging. The KT index, built around the knowledge translation framework on aging and health developed by the WHO [31], was operationalized through six items that measured two-way channels of communication between policymakers and researchers. Summative indices were calculated for each construct and were standardized to percentages. Accordingly, country rankings were generated and correlations made.

### Analysis

The indices were summarized by the median and the interquartile range. Correlations were generated using Spearman’s rank correlation (*r*_s_), and a series of bootstrap multiple linear regression models that tested the association between the enabling (institutional arrangements, knowledge creation) and mediating (knowledge translation) constructs with the outcomes (social and health policies and programs) was conducted. The standard error (SE), *p* value, and 95 % confidence interval for the regression coefficients were estimated using the bootstrap technique. The bootstrap is a non-parametric statistical technique that can provide more accurate inferences when the sample size is small. In the bootstrap analysis, 1000 samples of the same size as the original sample were drawn, with replacement from the original data set. This number of samples is needed for accurate confidence intervals [33]. Statistical analyses were performed using SPSS version 19 (Windows).

## Results

**Fig. 2:**
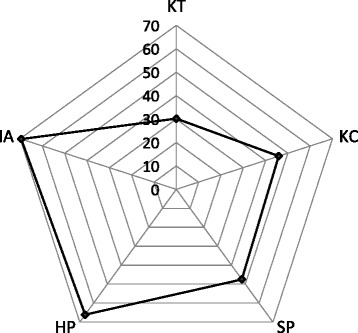
Overall scores in the institutional arrangements (*IA)*, knowledge creation (*KC*), knowledge translation (*KT*), and health and social policies and programs (*HP* and *SP*) constructs

Overall, findings indicate that countries scored highest on the IA index (median = 69.5), followed by the KC index (median = 45.9), and lowest on the KT index (median = 30.2). Also, health policies for older persons were more abundant than social policies, scoring 66.1 and 47.5, respectively (Fig. 2).

Country rankings are presented in Table 1 for each of the five indexes with 1 designated to the highest rank and 16 to the lowest in decreasing order of overall summative rank. Bahrain, Syria, Palestine, Lebanon, and Egypt ranked the highest, and Algeria, Iraq, Yemen, Djibouti, and Somalia ranked the lowest. Some inconsistencies were noted. Bahrain ranked low on KC despite accruing the highest rank on all other indices, which was mostly due to lack of accessible publications and national reports as well as the absence of research institutes on aging. However, the presence of these items in Yemen boosted its KC rank despite its low overall ranking. Findings for Algeria indicated that while it accrued a low overall ranking, its investment in KT was high relative to the region. This was owing to the production of policy briefs, a high level of communication between policymakers and researchers, and involvement of ministries and researchers in national research funding agencies.

**Table 1:** Country rankings by respective index and country-specific data on proportion of people older than 65 and GDP per capita

Countries	Overall ranking^a^	Enabling constructs		Mediating construct	Outcomes policies and programs		Country-specific data	
		Institutional arrangements (IA)	Knowledge creation (KC)	Knowledge translation (KT)	Social (SP)	Health (HP)	Percentage of population over 65 (2012) [2]	GDP per capita (international $) (2012) [36]
Bahrain	1	1	10	1	1	1	2.3	41,463
Syria	2	3	1	2	4	7	4.3	–
Palestine	3	2	2	5	2	6	3.1	4,929
Lebanon	4	5	2	3	6	1	8.8	16,931
Egypt	5	3	2	5	8	5	5.9	10,875
Jordan	6	7	2	10	3	3	3.6	11,544
Qatar	7	9	7	8	4	7	0.9	133,341
Oman	8	7	12	5	8	3	3.0	44,851
Libya	9	6	7	10	11	11	5.0	23,446
Morocco	10	11	9	12	13	7	5.1	6,898
Sudan	11	10	10	9	7	14	3.3	3,926
Algeria	12	13	13	4	12	13	4.7	13,008
Iraq	13	12	14	14	10	10	3.2	14,806
Yemen	14	14	6	13	14	12	3.0	3,833
Djibouti	15	15	15	14	15	15	4.1	2,857
Somalia	16	16	16	14	16	15	2.8	–
Median (out of 100)		69.5	45.9	30.2	47.5	66.1		
Interquartile range (IQR)		28, 82	29, 61	12, 48	30, 54	31, 74		

Note: percentage of population over 65 years is deflated in countries with very high non-national migrant worker populations (such as Bahrain and Qatar)

^a^Overall ranking is based on summative raw scores of the five constructs

The rankings were juxtaposed with two context-specific indicators: percentage of the population over the age of 65 and the GDP per capita for each Arab country. Oil-rich countries such as Bahrain, Qatar, and Oman in the Arab Gulf and Libya in North Africa, with markedly higher GDP per capita, did not necessarily receive a higher overall ranking, and thus displayed a weak correlation between a country’s wealth and its investment in population aging policies and programs. Similarly, the percentage of the older population did not necessarily harmonize with the overall ranking. For example, Algeria, with 4.7 % of its population older than 65, ranked 12th, while Palestine with only 3.1 % of its population older than 65 ranked third.

Correlations between the five indices are summarized in Table 2 and Fig. 1. Spearman’s correlations showed significant associations between all pairs of indices, except for between KC and KT (*p* value =0.054). IA showed the highest correlation with both KT and KC (*r*_s_ = 0.77, *p* value <0.001; *r*_s_ = 0.68, *p* value =0.004, respectively) and similarly high coefficients of correlation with SP and HP (*r*_s_ = 0.84, *p* value <0.001; *r*_s_ = 0.79, *p* value <0.001, respectively). Results from the bootstrap multivariable linear regression are summarized in Table 3. In the unadjusted models, the three indices showed significant associations with both SP and HP. Adjusting for KT, KC lost its significance while IA maintained it with both outcomes. Further adjustment for all the variables did not change appreciatively the results for IA but attenuated the association of KC with the outcomes. However, the association of KT with the outcomes lost its significance and was greatly attenuated from 0.64 to 0.08 for SP and from 0.78 to 0.12 for HP when adjusted for both IA and KC.

**Table 2:** Results of Spearman’s bivariate correlations

	Knowledge creation (KC)	Institutional arrangements (IA)	Knowledge translation (KT)	Social policies and programs (SP)	Health policies and programs (HP)
	*r*_s_ (*p* value)	*r*_s_ (*p* value)	*r*_s_ (*p* value)	*r*_s_ (*p* value)	*r*_s_ (*p* value)
KC	1.0	0.68 (0.004)	0.77 (<0.001)	0.84 (<0.001)	0.79 (<0.001)
IA		1.0	0.49 (0.054)	0.59 (0.017)	0.56 (0.023)
KT			1.0	0.69 (0.003)	0.64 (0.008)
SP				1.0	0.74 (0.001)
HP					1.0

**Table 3:** Results of the Bootstrap linear regression (*N* = 16)

	Dependent variable: social policies and programs (SP)								
	Unadjusted			Adjusted for KT			Adjusted for the remaining two constructs		
	Beta	SE	*p* value	Beta	SE	*p* value	Beta	SE	*p* value
KC	0.59	0.21	0.016	0.30	0.27	0.292	−0.15	0.20	0.376
IA	0.66	0.07	0.001	0.63	0.19	0.009	0.70	0.19	0.013
KT	0.64	0.19	0.011				0.08	0.21	0.671
	Dependent variable: health policies and programs (HP)								
	Unadjusted			Adjusted for KT			Adjusted for the remaining two constructs		
	Beta	SE	*p* value	Beta	SE	*p* value	Beta	SE	*p* value
KC	0.84	0.24	0.004	0.56	0.29	0.080	0.17	0.37	0.457
IA	0.76	0.12	0.001	0.69	0.22	0.010	0.60	0.30	0.042
KT	0.78	0.23	0.005				0.12	0.30	0.634

## Discussion

Findings from this study suggest a strong presence of state infrastructure and institutional arrangements on aging in Arab countries, and these constituted the most robust construct for the advancement of social and health policies and programs for older persons. In contrast, associations with the knowledge translation construct were overall weak, possibly indicating a deficiency in factors pertinent for promoting evidence-based decision-making and policy-relevant research.

**Fig. 3:**
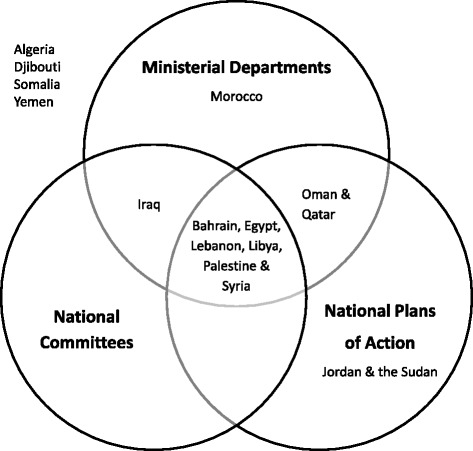
Institutional arrangements in the Arab region

Over the past 10 years, UN agencies have emphasized the need to establish specialized institutional arrangements within governments, and calls made by the MIPAA [32] and the Arab Plan of Action on Ageing (APAA) [34] in 2002 have influenced the establishment of ministerial departments, national committees, and national plans of action on aging in several Arab countries [4]. Further examination of the data indicates that these three MIPAA-designated formal arrangements on aging are found concurrently in 6 out of 16 countries (Bahrain, Egypt, Lebanon, Libya, Palestine, and Syria) and completely lacking in four (Algeria, Djibouti, Somalia, and Yemen) (Fig. 3). When prompted to identify challenges to implementation of evidence-based strategies and programs on aging, countries reported lack of financial resources and the need for evidence-based and research-informed guidelines for best practices as the main barriers. Interestingly, the least reported obstacle was perceived to be “political will.” These findings underscore the strong role of budding institutional arrangements on aging across the Arab region as integral conduits for the application of aging research to policy and practice and underline the importance of their reinforcement across all Arab countries.

In this study, the level of knowledge creation on aging was evaluated based on the availability of data on older persons, the production of national reports and scientific studies in the 5 years prior to the study, as well as facilitators for knowledge production such as the presence of research institutes and funding opportunities. Syria, Palestine, Lebanon, Egypt, Jordan, Yemen, Qatar, and Libya ranked highly in the production of aging knowledge due to the availability of recent age- and gender-disaggregated census data, national reports on aging, and aging research. Yemen ranked high on knowledge creation (sixth), largely due to the publication of national reports by the Yemeni Center for Social Studies and Labor Research and availability of research on aging. Almost half of the countries noted that they host institutes that undertake aging research (such as the Center for Studies on Ageing in Lebanon, the National Centre for Social Studies, and the Cairo Demographic Centre in Egypt). Additionally, Egypt, Morocco, and Qatar indicated that their national funding agencies allocate funds to aging research. Yet, knowledge production on aging remains low in Algeria, Djibouti, Iraq, and Oman, and health research capacity needs to be strengthened in countries where the proportion of older persons is rapidly increasing.

The very low median score of the KT construct in this study and its weak correlation with the outcomes (HP and SP) net of the effect of the other constructs suggest that knowledge translation in the field of aging is still at its infancy in the Arab region. These findings reflect an overall deficiency in the availability of data repositories, the production of policy briefs, as well as in communication channels between policymakers and researchers. Although KT studies on aging in the Arab region are lacking, a number of studies have been conducted to investigate the use of health systems and research in overall health policymaking [25, 27], thus allowing some comparisons to be made. For example, El-Jardali and colleagues [27] shed some light on the lack of utilization of scientific research in Yemen, noting that, to a larger extent than other countries, “scientific evidence [in Yemen] is not delivered at the right time and lack information on quality and local applicability.” These findings are consistent with the results from our study showing a low ranking for Yemen. Furthermore, our findings on Bahrain’s commitment to knowledge translation is corroborated by other studies in the region [25], despite the scarcity of aging research.

Other studies have been conducted in the region applying a pragmatic framework, the “policy-effectiveness feasibility loop” (PEFL) as a tool to strengthen linkages between researchers and policymakers and inform public policy in the areas of cardiovascular disease and diabetes [35]. PEFL integrated epidemiological modeling with policy situation analyses in order to generate feasible context-specific options that undergo evaluation and re-appraisal. Such knowledge translation initiatives strengthen evidence-based policymaking as well as policy-informed aging research and may well be relevant to knowledge translation in aging research.

### Limitations

The findings of this study need to be interpreted in light of some methodological limitations and offsetting strengths. Our study lacked data from 6 out of the 22 Arab countries, 3 of which are resourceful oil-rich countries (Kuwait, Kingdom of Saudi Arabia, and United Arab Emirates), which may have impacted country rankings. The data is cross-sectional in nature; policy formulation may have preceded knowledge creation and therefore, no causal relationships can be determined. The availability of publications on aging was based on national studies and did not include the more abundant small-scale academic research with stronger methodologies and analyses. Furthermore, responses were based on self-reports from country investigators and on document reviews, which do not necessarily reflect level of implementation and success on the ground. Yet, a multi-stakeholder approach was called upon in filling the questionnaire, with country investigators consulting focal points in ministerial bodies, UN agencies, NGOs, and medical schools. Also, the methodology was grounded in a rigorous evidence-based mapping of policies, programs, institutions, and data in the region, all of which strengthen the validity of the data collected.

The conceptual framework must also be considered in light of its own shortcomings. While the framework depicts knowledge creation and translation as enabling and mediating constructs, respectively, for social and health policies and programs, it does not encompass more complex interactions such as the influence of external and international agencies and situations where the pathway is reversed and where policy influences the creation of knowledge. Further empirical research with these models may shed light on alternate mechanisms.

## Conclusions

The mapping exercise provided a unique opportunity to concurrently examine the relationship between institutional arrangements and knowledge creation and translation in aging policies and programs in the Arab region. We have shown that, in the field of aging and in Arab countries, institutional arrangements are more strongly associated with policymaking and service provision than knowledge creation and knowledge translation, possibly indicating a deficiency in factors pertinent for promoting evidence-based decision-making and policy-relevant research. This warrants the need for strengthening institutional infrastructure in public agencies. Findings from this study could also act as a baseline for comparison with future studies in the aftermath of the socioeconomic changes and political transformations of recent years. These events will profoundly influence the political, economic, and research landscape of many Arab countries and are likely to trigger more knowledge creation but make knowledge translation more, rather than less, difficult in the future.

### Ethical approval

Ethical approval was not required.

## Additional file

Additional file 1:
**List of indicators of the five distinct constructs (IA, KC, KT, SP and HP).** (DOCX 29.2 kb)These five distinct constructs (IA, KC, KT, SP and HP) were operationalized into indices using several indicators of the yes/no, five-point likert-scale and open-ended type of questions. These are listed in Additional file 1 along with question types.

## Abbreviations

ESCWA: Economic and Social Commission of Western Asia
HP: health policies and programs
IA: institutional arrangements
ICPD: International Conference on Population and Development
KC: knowledge creation
KT: knowledge translation
SP: social policies and programs
UNFPA: United Nations Population Fund
WHO: World Health Organization
