# Supplementary material for: A survey of knowledge-to-action pathways of aging policies and programs in the Arab region: the role of institutional arrangements
Source: Implement Sci. 2015 Dec 11;10:170. doi: 10.1186/s13012-015-0360-8 (PMC4676156; doi:10.1186/s13012-015-0360-8)
Supplement: Additional file 1: — List of indicators of the five distinct constructs (IA, KC, KT, SP and HP). (DOCX 29.2 kb)These five distinct constructs (IA, KC, KT, SP and HP) were operationalized into indices using several indicators of the yes/no, five-point likert-scale and open-ended type of questions. These are listed in Additional file 1 along with question types. [file 13012_2015_360_MOESM1_ESM.docx]

**Additional Files**

| **Indicators for the *Institutional Arrangements* (IA) construct** | | |
| --- | --- | --- |
| Presence of IAs | Do you have a department/office/unit that caters to older population issues in your country? | None  Non-specialized  Specialized |
|  | Do you have a National Commission/National Committee that caters to older population issues in your country? | None  Non-specialized  Specialized |
|  | Does your country have a comprehensive stand-alone national policy/plan of action/strategy that specifically addresses ageing? | No/Yes |
| Effectiveness of IAs | Is the IA (above) intersectoral and includes representatives from ministries, civil society, academia, UN agencies and international agencies? | Yes/No for each item |
|  | To what extent is the IA in your country involved in advocacy, planning, implementation, resource/fund mobilization, research and data collection, advisory and technical support? | 5-point likert scale |
|  | How many of the following issues for older people are addressed in your country’s policies? (health coverage, pension plan, poverty reduction, income generation, on-job training, social coverage, literacy/education, housing, elderly neglect and abuse, disability, tax exemption, promoting positive image of ageing) | 0-2  3-5  ≥6 |

| **Indicators for the *Knowledge Creation* (KC) construct** | | |
| --- | --- | --- |
| Extent of KC | Since MIPPA 2002, have there been any national (country-wide) studies/reports specifically on ageing/older persons conducted by Governmental Agencies (such as by a Ministry, Bureau/Center of Statistics, National Commission)? | 0, 1 or 2, ≥3 |
|  | Since MIPPA 2002, have there been any national (country-wide) studies/reports specifically on ageing/older persons conducted by independent researchers in any of the following institutions/agencies: universities, NGOs, international organizations...? | 0, 1 or 2, ≥3 |
|  | Are there any data/studies on the following issues among the older population?   1. nutritional intake/malnutrition 2. mobility problems and physical disability 3. mental health needs | No/Yes |
| Facilitators for KC | When was the last time a census was done in your country? | No/Yes |
|  | Are reports produced from the census disaggregated by age and gender? | No/Yes |
|  | Are there any research institutes/centers/units within universities or any networks/working groups that focus on ageing research? | No/Yes |
|  | To what extent is ageing/older persons’ issues one of the priority themes/areas of the national research agenda/funding? | 5-point likert scale |
|  | Are there regular media programs (TV, radio show, newspaper columns…) that specifically address ageing and older people’s concerns? | No/Yes |

| **Indicators for the *Knowledge Translation* (KT) construct** | | |
| --- | --- | --- |
| Is KT a main obstacle for the implementation of the national strategy/programs on ageing? | | 5-point likert scale |
| Push Factors | Is there a repository where knowledge on ageing and older populations (reports, policy briefs, studies…) can be easily accessed by concerned stakeholders…? | None  Non-specialized  Specialized |
|  | Do researchers or intermediary organizations publish policy briefs/newsletters on ageing issues? | Never  Rarely  Occasionally  Often |
|  | To what extent do researchers communicate with policymakers to promote/advocate for ageing issues? (Such as periodic meetings, national committees…) | Never  Rarely  Occasionally  Often |
| Pull Factors | To what extent are policymakers involved in or part of the research conducted on older populations in the country? (Being co-investigators, members of steering committees of research…). | Never  Rarely  Occasionally  Often |
|  | Do national funding agencies for research include representatives from the following ministries:   1. Social affairs 2. Health 3. Other | No/Yes |

| **Indicators for the *Social policies and programs* (SP) construct** | | |
| --- | --- | --- |
| Ageing in Place | Are there any policies/programs that support/build capacity for caregivers? (e.g. training, respite services for caregivers, consultation services…) | No/Yes |
|  | Are there any other policies/programs that promote ageing in place within the home environment? (e.g. meals on wheels, discount on electricity and water, home management services…) | No/Yes |
|  | Are there any policies/programs that specifically target older people living alone? | No/Yes |
|  | Are there any policies/programs that protect older people who have no family or are homeless? | No/Yes |
|  | Are there any policies/programs/civil laws that address neglect, abuse and violence specifically towards older persons? (e.g. legal advice, hotline, providing food and shelter for older people who are victims of abuse…) | No/Yes |
|  | Are there policies/programs that promote intergenerational solidarity such as young adults acting as elder sitters, volunteers, and families hosting older people? | No/Yes |
|  | Are there any policies/programs that promote older people’s mobility outside the home environment? (e.g. discount on public transportation, safer roadways, public toilets, benches, tailored social and recreational activities, accessible public buildings…) | No/Yes |
| Self-Sufficiency | Are there any policies/programs that support income-generating projects (example microcredit schemes) for older people? | No/Yes |
|  | Are there policies that mandate social protection/pension for the elderly people in the informal sector? | No/Yes |
|  | Are there programs addressing older adults in rural areas? | No/Yes |
|  | Are there policies/programs that promote literacy among older populations? | No/Yes |
|  | Are there programs that promote lifelong learning programs/third age universities for older people? | No/Yes |
|  | Are there programs that promote the use of communication technology among older populations (for example computer training...)? | No/Yes |
|  | Are there interventions or programs that address ageism and discriminatory images of older people? (e.g. agencies that promote positive image of older people) | No/Yes |
|  | Do you have a national emergency preparedness plan that is specialized for older persons? | None  Non-specialized  Specialized |
|  | Are there any programs that target older population groups among refugees? | No/Yes |
|  | Is there any evidence of mainstreaming elderly issues in disaster relief programs? Example: capacity building of disaster relief workers to care for older people. | No/Yes |
| Nursing and elderly homes | Are the services of elderly homes, in general, affordable? | No/Yes |
|  | Are there any standards of care/accreditation guidelines for nursing homes and day care centers in the country? | None  In Progress  Finalized  Implemented |

| **Indicators for the *Health policies and programs* (HP) construct** | | |
| --- | --- | --- |
| Primary Prevention | Are there any policies/programs that promote healthy ageing:   1. smoking cessation 2. physical activity 3. screening for breast cancer 4. screening for diabetes 5. screening for hypertension | Planned  Implemented  Sustainable |
| Access to Services | Are there any policies/programs that facilitate access to health care services that explicitly target older populations? (e.g. primary health care, free medications, transport to health care facilities) | No/Yes |
|  | Are there any NGOs/units/organizations that provide end of life/palliative care services at home? | No/Yes |
|  | Are Alzheimer, dementia and other mental health conditions related to older people covered by public health insurance schemes? | No/Yes |
|  | Are there any programs that provide mobile clinics for the frail/disabled elderly living at home? | No/Yes |
|  | Are there programs that provide meals on wheels? | No/Yes |
| Training programs on geriatrics and gerontology | Are there training programs to health and social workers regarding elder abuse and detection? | No/Yes |
|  | Does your country have the following programs:  Geriatrics curriculum in Medical Schools?  Geriatrics fellowships/residencies in hospitals?  Geriatric module in family medicine training?  Geriatrics integrated in already existing courses in Medical Schools? | No/Yes |
|  | Does your country have the following programs:  Gerontology programs to nurses?  Gerontology programs to social workers?  Gerontology programs to occupational and physical therapists? | No/Yes |
|  | Does your country have the following programs for healthcare providers:  Ad-hoc short courses/diplomas in geriatrics or gerontology?  Ad-hoc training to workers in Primary Health Care Centres in geriatrics and gerontology? | No/Yes |
